# Theory of the remission process of schizophrenia (Nakai)

**DOI:** 10.1002/pcn5.96

**Published:** 2023-05-21

**Authors:** Minoru Sugibayashi

**Affiliations:** ^1^ Department of Psychiatry, Aijinkai Healthcare Corporation Takatsuki General Hospital Osaka Japan

**Keywords:** critical period, naturalistic approach, patient's drawing, progress chart, schizophrenia

## Abstract

Hisao Nakai (1934–2022) surprised many experts with his prolific publication of papers ranging from meticulous therapeutic engagement and observations to original research. The “theory of the remission process of schizophrenia” is a representative example of Nakai's research and theories. The significance of Nakai's theory of the remission process is that it let the world know about the existence of schizophrenia's remission process, which had not been previously recognised. Additionally, Nakai's discovery of the “critical period” was significant and his detailed description of the same, which marks the beginning of the remission process, is widely considered to be fundamental. Nakai closely followed common phenomena that appeared accidentally, and he plotted the reactions of the autonomic nervous system, patient's dream content, and drawing content on a chart to show, for the first time, the clear emergence of a “critical period,” and demonstrated the crucial role of the remission process. Nakai explained how the process progresses sequentially from the “critical period” to the “early remission period” and the “late remission period.” He identified many specific indicators and discovered a great significance in innovative therapeutic engagement in each of these periods. Nakai's findings are supported by meticulous clinical observations and are free from simplifications that stem from simple theorization. In particular, close observation of the remission process was conducted with full engagement and concern of the therapist, without which, the remission process would not have been visible.

## Graphical abstract

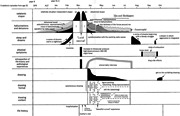

## INTRODUCTION TO HISAO NAKAI

Nakai was born in Japan in 1934. Five years later, World War II broke out, engulfing the entire world in war. Nakai was 11 years old when Japan surrendered in 1945. In 1959, he graduated from the Faculty of Medicine, Kyoto University, and in 1960 he became a research assistant at the Institute for Virology at the same university (now the Institute for Life and Medical Sciences). In 1966, he decided to become a psychiatrist and worked at the Department of Neuropsychiatry of the University of Tokyo Branch Hospital and Aoki Hospital, a private psychiatric hospital. Using the results of his creative clinical innovations, Nakai published many pioneering papers on the therapeutic approaches to schizophrenia. Clinicians referred to him as a genius. He contributed greatly to the history of psychiatry in Japan. Later, he served as an associate professor at the Department of Psychiatry of the Nagoya City University School of Medicine, and as a professor at the Department of Psychiatry and Neurology in the Kobe University School of Medicine. He died on August 8, 2022, aged 88 years.

In the 1970s and 1980s, Japanese psychopathology made great progress.^1^, ^2^, ^3^, ^4^, ^5^, ^6^, ^7^, ^8^, ^9^, ^10^, ^11^, ^12^, ^13^, ^14^, ^15^, ^16^ Nakai, Yomishi Kasahara (1928–), Hiroshi Yasunaga (1929–2011), Tadao Miyamoto (1930–1999), Bin Kimura (1931–2021), and others were the forerunners of psychopathology in Japan. Kasahara^17^ received attention for his research on student apathy, and Yasunaga^18^, ^19^ built a unique theory (phantom space theory) that could explain the experience structure of schizophrenia in a unified manner. Miyamoto^20^, ^21^ broadly expanded psychopathology to the theory of civilization, while Kimura^22^ developed his own theory of self and life based on “between‐ness(aida).”

Among Nakai's many achievements,^23^ the most prominent ones are listed below.

### The theory of the course of schizophrenia and its therapy

Nakai surprised many experts with his prolific publication of papers ranging from meticulous therapeutic engagement and observations to highly original research. The “theory of the remission process of schizophrenia” is a representative example of Nakai's research and theories.^24^

In his study, Nakai examined the use of drawings in psychotherapy for patients with schizophrenia. It was shown that the feature of the psychological space of schizophrenia changes following a change in drawing. Consequently, the remission process of schizophrenia, which has been overlooked in the past, has been described in detail in his work. Nakai referred to French surgeon Henri Laborit's theory^25^ about the biological response to invasion and highlighted the deep correlation between the body and the mind.

According to Matsumoto,^23^ Nakai was influenced by Laborit. Laborit discovered that the biological reaction after major surgical invasion occurs as series of events oscillating between parasympathetic and sympathetic dominance. Laborit refers to this alternating response as the “post‐operative oscillatory response.”

### Translation of Sullivan's research

In an era when antipsychotic drugs had not yet been developed, American psychiatrist Harry S. Sullivan^26^, ^27^, ^28^, ^29^ attracted a great deal of attention in Japan when he achieved nearly 70% social remission in patients with schizophrenia in an acute ward program designed by him. However, Sullivan's texts were extremely difficult to understand and even native English speakers could not comprehend them. Nakai translated most of Sullivan's writings into easy‐to‐understand Japanese. He also introduced Sullivan's perspectives and clinical practice models to Japanese psychiatric professionals.

### Central role in providing mental healthcare for victims of the Great Hanshin Earthquake (1995)

When the Great Hanshin Earthquake struck Kobe, where Nakai worked, he quickly led the charge and served as a coordinator. He developed outreach activities for the victims and supported their mental health. This was the first full‐scale practice of disaster psychiatry^30^ in Japan which served as a model for the construction of subsequent support systems during other disasters. Not only did Nakai translate several American texts on the theory of psychological trauma, he also described the enlightening theories of psychological trauma that were based on clinical sensibilities and refrained from the inflammatory discourse.

### Translation of modern Greek poetry

As a demonstration of his outstanding language skills and poetic sensibilities, Nakai translated modern Greek poetry, like that by Constantine P. Cavafy, for which he won high praise. Nakai won the Yomiuri Prize for Literature in 1989 and the Greek Translation Prize for Literature in 1991.

## INTRODUCTION TO THE THEORY OF THE REMISSION PROCESS OF SCHIZOPHRENIA

Nakai's theory of the remission process was published in the article, “Remission process from the state of schizophrenia: Longitudinal observations through psychotherapy combined with drawing” (1974).^31^ The following sections provide an overview of all nine sections, from the Introduction to the Conclusion of Nakai's article.

### Introduction

The onset of schizophrenia is often verbally expressed by patients, but accounts of the remission process of schizophrenia are scarce. Poor verbal productivity was observed to be a negative symptom. However, the remission of schizophrenia is an eventful process that follows a definite, sequential, and stepwise course. This can be seen, for example, by recording nonspecific events, like sudden diarrhoea or constipation, unexplained fever, dizziness, and epigastric discomfort, and plotting them on longitudinal and multidimensional charts to correlate them with the patient's expressive activity and behavioural characteristics. The remission process of any ailment is one in which minute, one‐off events and changes must always be observed in the context of the entire process. The patient's drawings under psychotherapeutic engagement and verbal exchanges between the patient and the therapist mediate this activity, which is useful in grading this remission process.

It is crucial to note that the remission process is not the reverse of the onset process. The former must be pursued using different logic. Events that are considered aberrant at the onset of the pathology may have other implications in the remission process.

### Methodological development

Nakai's principles of the nonverbal management approach to schizophrenia are described in the sections below.

(a) Nakai aimed to enable some form of psychotherapeutic approach for all patients with schizophrenia, therefore he focused on approaches that were inclusive of patients who faced difficulties in a verbal approach. For this, he developed and utilized several auxiliary techniques, including drawings.
(b) Nakai encouraged drawing only in cases where a therapeutic relationship was established. All drawings were made under “participant observation” (Sullivan)^26^ or “observation while engaging,” in other words.
(c) Drawing was strictly a means of communication, and judgments based on aesthetic detection were rejected.
(d) Drawing was always positioned longitudinally in the “flow of therapy.” Nakai emphasised verbal and nonverbal interactions with the patient rather than therapeutic interpretations from the clinician's perspective.
(e) The general principle of drawing is that it should be as nonmandatory as possible.

### The onset process of acute schizophrenia

Klaus Conrad served as a German Wehrmacht physician in the early stages of World War II. He was also a psychiatrist who described the early stages of schizophrenia among Wehrmacht soldiers.^32^ Nakai highly praised Conrad's research. Conrad used the term “*Trema*” to describe the pre‐onset state of acute schizophrenia, and Nakai expressed this term in Japanese as “*Aseri*” (lit. “impatience”). It is impossible to suddenly develop schizophrenia from a state of “*Yutori*” (lit. “comfort”). The pre‐onset state of schizophrenia involves first the period of “*Muri*” (lit. “overburdening”), followed by a gradual increase of “impatience.” Incidentally, even patients who are in a serious state of “incoherence” immediately understand the words “impatience” and “comfort,” and they often admit that they are in a “mess of impatience.” Furthermore, patients generally understand the feeling of “impatience” and using the term helps them in making others understand them or their condition.

A characteristic of the “impatience” sensation that precedes schizophrenia is that it is closely related to poor ability to localise the problem. Patients with schizophrenia tend to perceive small problems as big problems, and they may simultaneously try to solve all their problems. Contrastingly, patients with neuroses involve neurotic solutions that localise large problems into smaller problems using strategies like repression, displacement, and projection. Autonomic symptoms like headache, palpitations, and insomnia can also occur. Normally, such symptoms compel rest, but this does not serve as an effective warning during the pre‐schizophrenia stage.

### Acute schizophrenic state

Nakai summarised Conrad's description of the psychopathology of acute schizophrenia in a semiotic manner, and organised it by using the concept of semiotic contradiction.

However, Nakai did not stop at merely demonstrating a pathological understanding. For patients in an acute schizophrenic state, their disease shows subtle fluctuations “as though the wind is breathing.” During these subtle fluctuations, Nakai discovered a potential therapeutic approach. Even when a patient is in a state of the most severe catatonic agitation, verbal communication can be provided according to the pace of the patient's breathing, as though moving from a boat to a ship that rises and falls on violent waves. In doing so, it is possible to calm agitation.

Some patients draw spontaneously, but it is generally not possible to promptly introduce a drawing as a therapeutic setup. As was done by Gertrud Schwing,^33^ psychotherapy at this stage needs to begin with the therapist gently ensuring their physical presence next to the patient. Schwing emphasised a “maternal aspect.” This does not refer to the mother who takes a blunt approach, but rather the resolute mother depicted in the *Die Aufzeichnungen des Malte Laurids Brigge* (*Malte's Journal*) by Rainer M. Rilke. This mother tells the child who is afraid of the dark, “Do not be afraid, it is I who is the dark.”

### The critical period

The essence of Nakai's theory of the remission process of schizophrenia was his discovery of the “critical period.”

The critical period refers to a series of observed events that signal the termination of the schizophrenic state and transition to a remission process. It is generally noted that during the critical period, all the transient phenomena appear rather abruptly. In patients with schizophrenia, compulsive repetitive processes recede into the background and sequential processes move into the foreground. Capturing these requires close observation and the recording of seemingly accidental and mundane phenomena.

Importantly, it is interesting to note that there exists a nonharmonic activity in the autonomic nervous system of humans. Specific examples include alternating diarrhoea and constipation, unexplained fever, dizziness, epigastric discomfort, abdominal pain, and a burning physical sensation. Drug‐induced side effects may also be temporarily enhanced, physical diseases (e.g., appendicitis and trauma) may develop, and fainting attacks that may be confused with epilepsy may occur. Consequently, complaints of physical symptoms may increase, but they could be misunderstood as hypochondriac symptoms or agitation, and as an exacerbation of symptoms.

Patients begin to report that dreams as auditory hallucinations and delusions abate; however, on entering the critical period, the dreams may become intense nightmares that involve the autonomic nervous system. Additionally, the connection between emotions and the body is restored and the feeling of “impatience” is relieved in the remission period. A patient described this as “enough comfort to be self‐aware of my impatience.”

In the process of remission, it is noted that the ability to introspect and reminisce also reappears. Conrad indicated that the process in which patients vividly narrated morbid experiences lies within this critical period. Given that this critical period is extremely psychologically unstable and is related to a high risk of suicide, its “discovery” should not be a source of rejoicing. The early stage of the critical period particularly is also one of “terrifying intuition,” as the drawings clearly show. Various secondary problems emerge when the schizophrenic world is dismantled. For example, feelings of pressure may suddenly disappear. Patients often perceive a loss of ego boundaries rather than a sense of regained freedom in their behaviour and experience. This creates a sense of “soft superiority.” Psychiatrists should not suspect megalomania and should not attempt to repeatedly confirm this. As long as these actions are not taken, these thoughts will likely not become fixed, as in megalomania.

Self‐perceived body images that regenerate during the critical period are often monstrous or inorganic, and transient.

The critical period can be divided into early and late stages, from spontaneous to “projective” drawing, like “scribbling drawing.”^34^ The early period is further divided into the “terrifying intuition period” and the subsequent “chaotic or cosmic drawing period.” Moreover, the “explaining hallucinations and delusions period” tends to occur before or after the “chaotic or cosmic drawing period.”

The “terrifying intuition period” is drawn in such a way as to express the crisis of existence in a single picture. Examples include a “person underground, buried in rocks, and unable to move,” or “a cliff destroyed by waves and a dead tree on it.” They are often drawn with a striking touch that evokes deep emotions.

The “chaotic or cosmic drawing period” is drawn with images such as “an atomic bomb explosion” or “a gunman shooting a pistol,” with some depictions involving cosmic elements, such as “the solar system.” Commonly seen images in the intermediate period between the two drawing periods are “rivers without shores,” “bottoms of the sea,” and “countless soap bubbles.” These are borderless expanses, but they contain content and movement, and are not vague entities.

The “explaining hallucinations and delusions period” often expresses the experience of the preceding acute phase with outstanding ideas. Examples include the “course of a ball that bends subtly with the waves from people passing each other.”

The late stage of the critical period involves the “serene period” and the subsequent “regression–regeneration period.”

The “serene period” involves the feeling of a clearing a crisis. Conversely, colours feel bright and free, and they evoke a feeling of pure catharsis in the viewer. Images include a “sea where a storm is leaving” or the “morning sun shining through the clouds.”

The subsequent “regression–regeneration period” is simpler and more repetitive. Images such as “a baby wrapped in maternity clothes,” “mother and child,” a “chick hatching from an egg,” and a “snake” are repeatedly drawn. Another popular image is a “snail with horns.”

However, there was no repetition of the same object in the drawing during the critical period. Even “the tree,” which generally does not change much, changes every time it is drawn during the critical period.

The critical period is an opportunity for psychotherapy, which must be delivered at exceptionally high intensity. This critical period likely acts as a “potential barrier” during the transition from “schizophrenic pseudo‐homeostasis” to “normal homeostasis.” The difficulty in overcoming this barrier is one of the major causes of chronic schizophrenia.

Antipsychotic drugs are useful in triggering the critical period in patients with schizophrenia; however, psychological approaches can also trigger the critical period in them. Like the “potential barrier” in atomic physics (quantum mechanics), a special mental energy is required for overcoming the critical period. Some patients may remain in the middle of the critical period and experience distressing mental phenomena for years.

However, it is noted that once the critical period has passed, phenomena from the acute schizophrenic state do not easily reappear. If they do, then they must go through a certain preparatory state, which would be a new “recurrence” of the acute phase.

The experience of the critical period in such patients is perhaps more intense than in the acute schizophrenic state. In this stage, the patient experiences extreme loneliness, making it difficult to tide over a critical period without therapeutic support. Patients often seek death in such a situation and repeatedly attempt suicide. However, loneliness in this period is different from that in the acute period, and it is open to the human world. Physical disturbances during this period lead to the “wisdom of the body” that the patient should follow. This critical period provides the greatest therapeutic opportunities.

**Figure 1:**
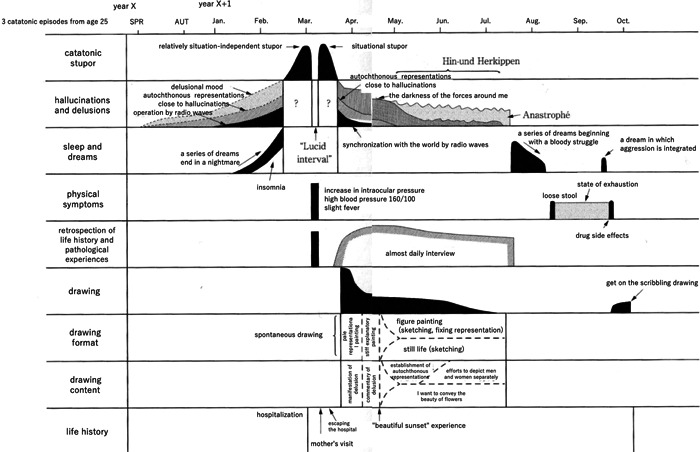
Course of a 38‐year‐old patient with schizophrenia. The patient had a catatonic course. Only the acute to critical periods are shown here.

**Figure 2:**
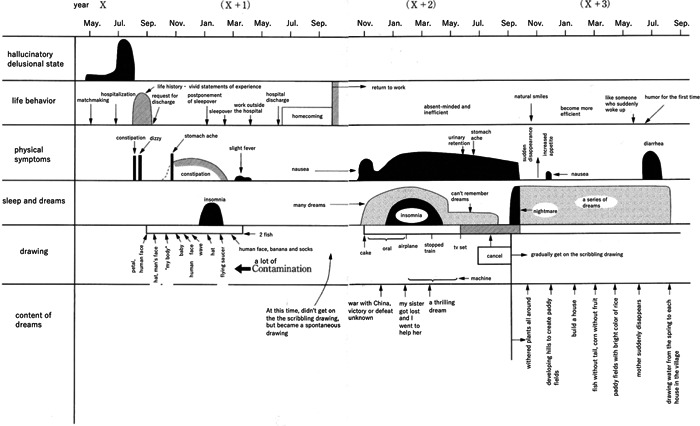
A paranoid course in a 28‐year‐old male with schizophrenia. The acute period to the end of the critical period is indicated.

**Figure 3:**
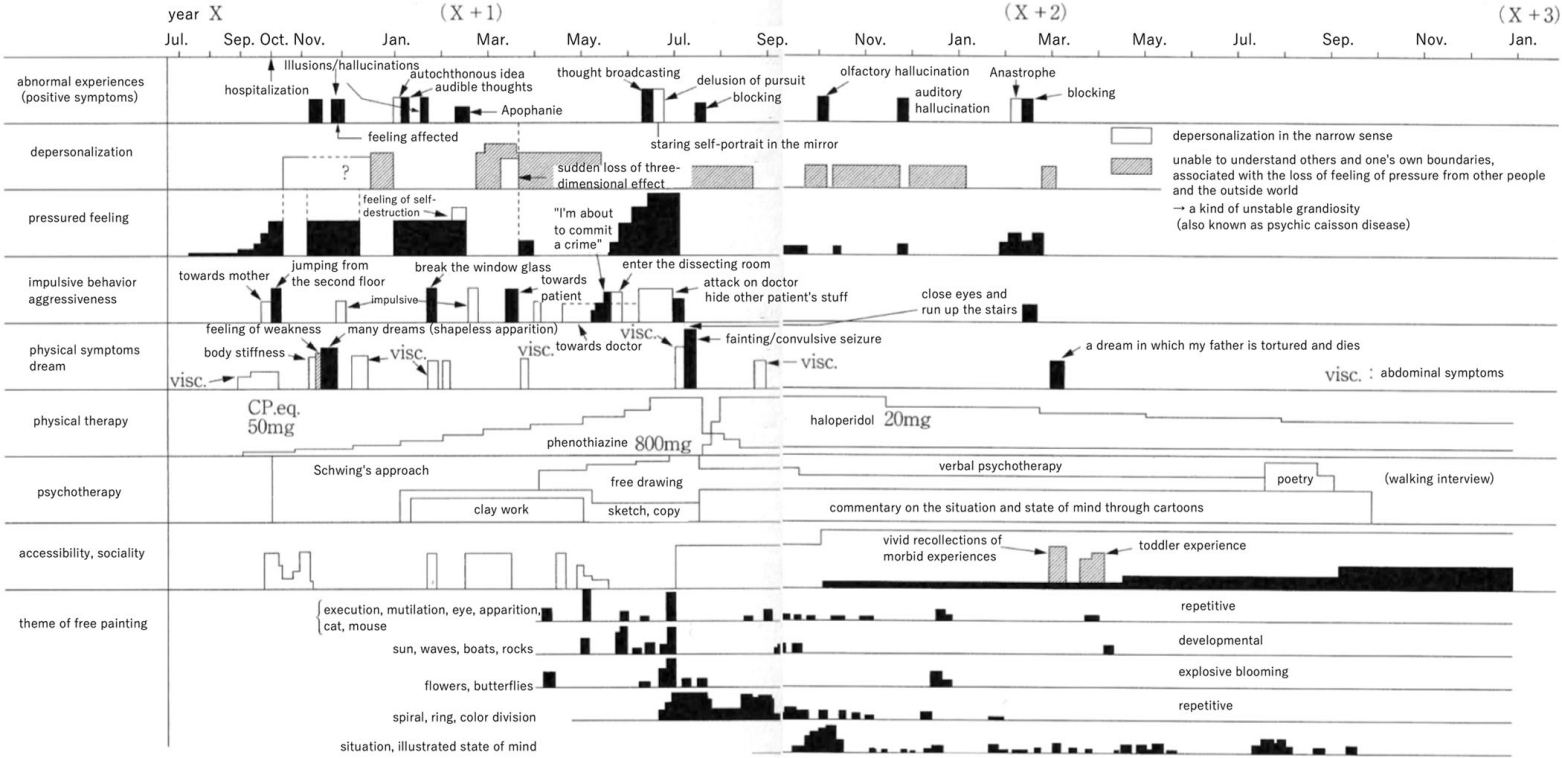
A disorganised course of a 21‐year‐old male with schizophrenia.

**Figure 4:**
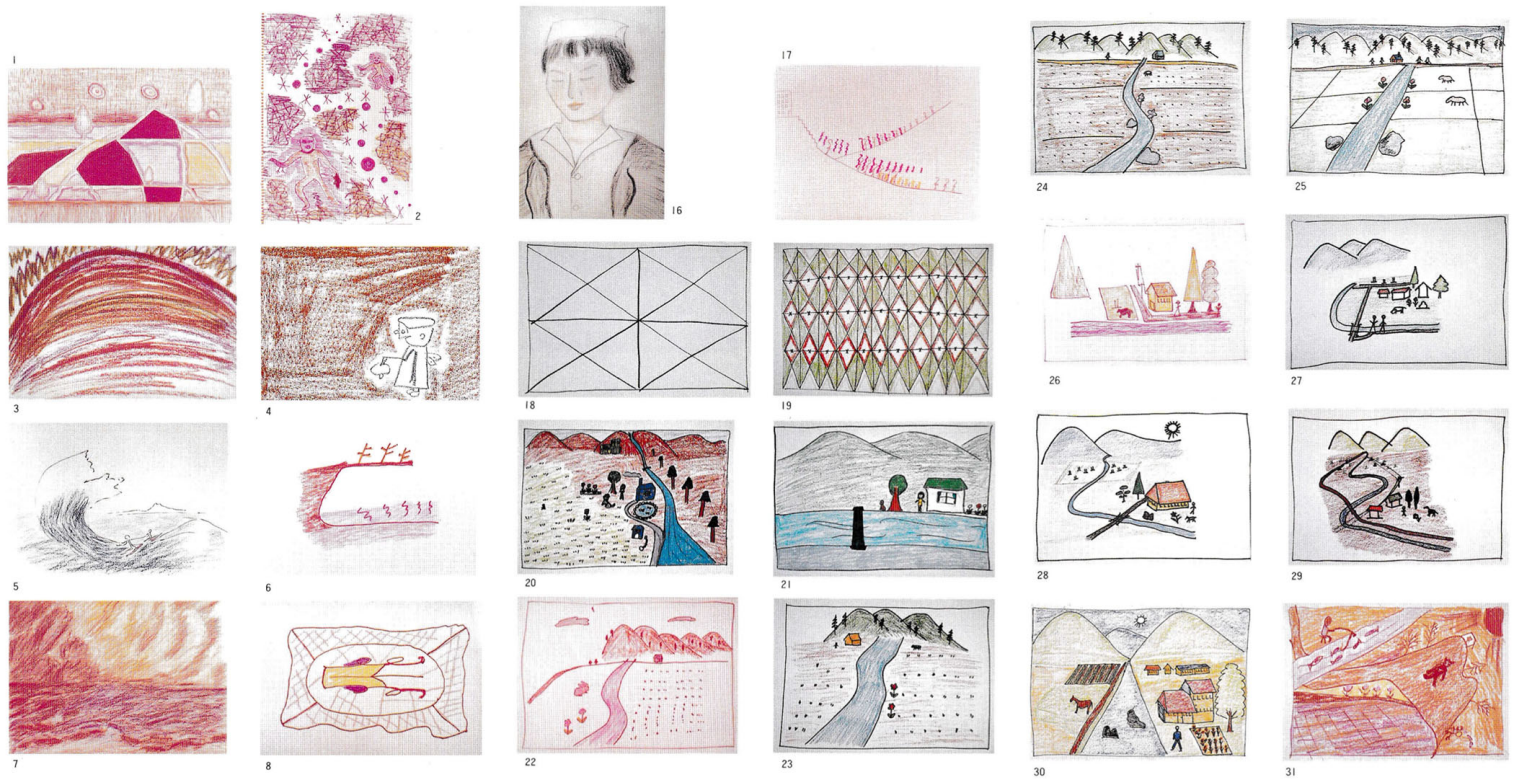
Twenty‐four out of 34 drawings by patients with schizophrenia with commentary, including drawings other than the remission process mentioned in Nakai's book.

In Nakai's study, detailed charts showed the critical period of 11 patients with schizophrenia. Among them, one case each of the catatonic (Figure 1), delusional (Figure 2), and disorganised types (Figure 3) is translated and reprinted here. Furthermore, some of the patients’ drawings at the time were published in later works.^35^ Partial reprinting of these drawings is provided in this paper from these sources (Figure 4).

Although Figures 1, 2, 3 are very detailed and minute, as in Nakai's original work, these figures have a significant impact on readers. It is possible to arrange these figures to make them easier to read but considering that the impact of the figures would be lost, the author chose to use Nakai's original figures and translate only the characters into English. Fortunately, because this is an electronic journal, readers will be able to read the details by enlarging the images. For supplementary purposes, the tables of words written in each figure are shown (Tables 1, 2, 3).

**Table 1:** Words written in Figure 1, a

	3 catatonic episodes from age 25
	year X year X + 1
	SPR AUT Jan. Feb. Mar. Apr. May. Jun. Jul. Aug. Sep. Oct.
catatonic stupor	relatively situation‐independent stupor
	situational stupor
	Hin‐und Herkippen
hallucinations and delusions	delusional mood
	autochthonous representations close to hallucinations
	operation by radio waves
	autochthonous representations close to hallucinations
	the darkness of the forces around me
	Anastrophe
sleep and dreams	a series of dreams end in a nightmare
	insomnia
	“Lucid interval”
	synchronization with the world by radio waves
	a series of dreams beginning with a bloody struggle
	a dream in which aggression is integrated
physical symptoms	increase in intraocular pressure
	high blood pressure 160/100
	slight fever
	loose stool
	state of exhaustion
retrospection of life history and pathological experiences	almost daily interview
	drug side effects
drawing	get on the scribbling drawing
drawing formats	spontaneous drawing
	pale representational painting
	stiff explanatory painting
	figure painting (sketching, fixing representation)
	still life (sketching)
drawing content	manifestation of delusion
	commentary of delusion
	establishment of autochthonous representations
	efforts to depict men and women separately
	I want to convey the beauty of flowers
life history	hospitalization
	mother's visit
	escaping the hospital
	“beautiful sunset” experience

a The words written in Figure 1 are shown here.

**Table 2:** Words written in Figure 2, a

	year X （X＋1） （X＋2）
	May. Jul. Sep. Nov. Jan. Mar. May. Jul. Sep. Nov. Jan. Mar. May. Jul. Sep. Nov. Jan. Mar. May. Jul. Sep.
hallucinatory delusional state	
life behavior	matchmaking hospitalization
	life history・ vivid statements of experience
	request for discharge postponement of sleepover sleepover
	work outside the hospital hospital discharge homecoming
	return to work absent‐minded and inefficient natural smiles
	become more efficient like someone who suddenly woke up
	humor for the first time
physical symptoms	constipation dizzy stomach ache constipation slight fever
	nausea urinary retention stomach ache sudden disappearance
	increased appetite nausea diarrhea
sleep and dreams	insomnia many dreams insomnia
	can't remember dreams nightmare a series of dreams
drawing	petal,human face hat, man's face “my body” baby
	human face wave hat flying saucer
	human face, banana and socks 2 fish a lot of contamination
	cake oral airplane stopped train
	tv set cancel machine gradually get on the scribbling drawing
content of dreams	At this time, did not get on the the scribbling drawing, but became a spontaneous drawing
	war with China, victory or defeat unknown
	my sister got lost and I went to help her
	a thrilling dream
	withered plants all around
	developing hills to create paddy fields
	build a house
	fish without tail, corn without fruit
	paddy fields with bright color of rice
	mother suddenly disappears
	drawing water from the spring to each house in the village

a The words written in Figure 2 are shown here.

**Table 3:** Words written in Figure 3, a

	abnormal experiences (positive symptoms)
	year X （X＋1） （X＋2） （X＋3）
	Jul. Sep. Oct. Nov. Jan. Mar. May. Jul. Sep. Nov. Jan. Mar. May. Jul. Sep. Nov. Jan.
abnormal experiences (positive symptoms)	hospitalization Illusions/hallucinations autochthonous idea
	audible thoughts Apophanie thought broadcasting
	delusion of pursuit blocking olfactory hallucination
	auditory hallucination Anastrophe blocking
depersonalization	feeling affected
	sudden loss of three‐dimensional effect
	staring self‐portrait in the mirror
	depersonalization in the narrow sense
	unable to understand others and one's own boundaries, associated with the loss of feeling of pressure from other people and the outside world→a kind of unstable grandiosity (also known as psychic caisson disease)
pressured feeling	feeling of self‐destruction
	“I'm about to commit a crime”
impulsive behavior aggressiveness	towards mother jumping from the second floor
	impulsive break the window glass
	towards patient enter the dissecting room
	attack on doctor hide other patient's stuff
	close eyes and run up the stairs
physical symptoms dream	visc. body stiffness feeling of weakness
	many dreams (shapeless apparition) visc. visc.
	towards doctor visc. fainting/convulsive seizure visc.
	a dream in which my father is tortured and dies
	visc.: abdominal symptoms
physical therapy	CP.eq. 50 mg phenothiazine 800 mg haloperidol 20 mg
psychotherapy	Schwing's approach clay work free drawing
	sketch, copy verbal psychotherapy
	commentary on the situation and state of mind through cartoons
	poetry (walking interview)
accessibility, sociality	vivid recollections of morbid experiences
	toddler experience
theme of free painting	execution, mutilation, eye, apparition, cat, mouse
	sun, waves, boats, rocks flowers, butterflies
	spiral, ring, color division situation, illustrated state of mind
	repetitive developmental explosive blooming repetitive

a The words written in Figure 3 are shown here.

### Early remission period

During the remission period, it was noted that the disharmony of the autonomic nervous system subsided, but vagal symptoms sometimes occurred. There is a perceived feeling of exhaustion and difficulty in concentrating, with an obvious weight gain. It is easy to suspect that these are negative symptoms, but in many cases the patients themselves feel a sense of relief. However, it is also a period in which complex human relationships can feel like a hassle, and where even thinking about the future and remembering the past remains painful.

At this stage, patients often experience a “cocooned feeling.” This represents a mild sense of detachment from internal and external events. Events feel like they are happening far away or underwater. Patients often report “a protective feeling.” It may appear to be a blunted affect, but rather it is the opposite, the beginning of localised emotional regeneration.

During localised emotional regeneration, the patient's dream content changes. The content changes from almost all nightmares to integrative content, conforming to the principles of reality. For example, there was a patient who began with “a dream in which the patient was looking for grass buds somewhere in a dry field,” followed by “a dream in which a hill was being cleared to create a rice field,” “a rice field changed to a fine colour that reflected a harvest,” and finally, a dream in which “there was a clear spring, and where water channels were laid to distribute the spring water to each house in the village.” Here, we can see the regeneration of the kairological time.

At this point, each of the characteristics of the delusional and disorganised types begin to manifest in the patients’ drawings. However, as remission progresses, the pathological characteristics begin to weaken.

Despite appearing stagnant at the first glance, a positive attitude toward drawing, differentiation of colours and forms, and verbal exchanges about the drawings generally increase. Therefore, therapists should not be misled by the lack of verbal exchange and awkward social behaviours during this period. During this period, patients will lose a sense of their internal rhythms if the environment forces them to engage in specific social behaviours. The patients become slaves to chronological time, and they become unable to act without external compulsion. Each patient went through the remission period at their own pace. One tip for safely enduring this period is to proceed according to the patient's pace of therapy.

Near the end of this period, the drawings began to change from repetitions of the same representation to a symbolisation of the same theme in a wide variety of ways, which increased the degree of freedom and clarified the central theme. This period is termed as the “progressive period” following the “cocoon period.”

### Late remission period

The functions of the autonomic nervous system gradually become harmonious and diurnal differences also appear. The patient's sense of exhaustion and difficulty in concentrating suddenly disappears. Moreover, the patient gives the impression of a person suddenly awakening to those around them.

One of the major indicators of the late remission period is recovery of sense of the season. The patient says things like, “I can feel spring for the first time in years.” Patients may try to retrospect and predict the future in the context of the present. They see the past as a single, continuous narrative. A greater sense of relief is created, and they can maintain this sense of relief even in the presence of others. They can also handle certain emergencies. Furthermore, their dream functions recover to a level similar to that of a healthy person. Verbal activities become progressively more active, and heuristic use of language becomes possible.

Regarding the drawings, the scribbles involve various developments around a central theme, but the patient begins to gradually lose interest in the drawings themselves, and the drawings that they compose may become mediocre. Contrastingly, the landscape montage technique (a technique developed by Nakai) changes rapidly. Consistency is restored, and the foreground has rich symmetry.

Therefore, attention must be paid to the prevention of recurrence. Measures like forcibly remodelling the patient's original character are not effective. It is thus ideal to utilize the heightened susceptibility common among individuals with schizophrenia. In the case of recurrence, subtle and indescribable sensations appear to precede the onset of delusional mood, and some patients can avoid recurrence by quickly perceiving these sensations.

Patient‐specific difficulties in interpersonal relationships can be long lasting. In reality, people generally live by cleverly organising and utilizing what is available to them. Contradictions and conflicts must somehow be sublimated toward integration. One characteristic of the Japanese society is that people must “be thoughtful.” To be thoughtful requires the accumulation of small psychological and practical innovations in dealing with disagreements and conflicts. If the surrounding people, to help the patient to overcome these aspects, make the patient feel “impatient,” it can result in a relapse. It is thus safer to understand patients’ psychological characteristics and to use them strategically in social situations. In other words, there are two strategies: maintaining psychological distance (spatial strategy) or taking a grace period (temporal strategy). Still struggling to localise problems, patients often are immediately forced to revise their overall picture of the world when they encounter a surprise event. The same applies when being forced to make uncertain (especially speculations about interpersonal relationships) and long‐term predictions. To cope with this, the patient may maintain a psychological distance by reducing the event to as small and insignificant as possible or leaving the problem aside for the time being and postponing the solution to secure a temporal grace period.

### Chronification

Schizophrenia can become chronic at any stage of life. The disease can become chronic in the acute schizophrenic state, as well as at all stages during the remission process. It can even become chronic during the unstable state, which is a critical period. Even the pre‐onset state can change to chronic.

If the remission process is characterized by a tendency toward a semantic acquisition, affinity to the therapeutic set‐up, sequential development, and so on, then the chronic process can be defined as a tendency toward meaninglessness, avoidance of the therapeutic set‐up, and compulsive repetitions. In the process leading to the chronic state, the therapeutic relationship inevitably disappears sometime during the process. From the therapist's perspective, this is experienced as *fadenverlieren* (i.e., the loss of the “guiding thread” of the therapeutic relationship”). Even the therapeutic interactions mediated by drawings may be temporarily interrupted during this stage.

Almost all phenomena in the critical period appear when a chronic state is resolved. Presumably, many patients with chronic conditions enter remission through recurrence of acute schizophrenic conditions.

### Conclusion

The above‐mentioned discussion is not the natural course of schizophrenia remission, but a complex process in which patients with schizophrenia and the therapist interact, and physical therapy and psychotherapy proceed together. This formulation is based on the concept of remission. The framework and milestones of therapy are also discussed.

## SIGNIFICANCE OF THE THEORY OF REMISSION PROCESS

Psychopathology, with its excessive focus on the pathology and abnormalities of schizophrenia, has not paid much attention to the disease onset process, how the pathology begins and develops from a healthy state, or on the remission process^31^ studying how the diseased state recovers to a healthy state.

Conrad provided a detailed description of the onset process; however, no research has focused on the remission process of schizophrenia. The significance of Nakai's theory of the remission process is that it made known to the world the existence of the remission process, which had not been previously recognised. Additionally, the discovery of the “critical period” is significant as it marks the beginning of the remission process. Nakai closely followed the common phenomena that appeared accidentally, and he plotted the reactions of the autonomic nervous system, the patient's dream content, and the patient's drawing content on a chart to show for the first time the clear emergence of a “critical period,” and demonstrated the crucial role of the remission process. This indication could be retested immediately on patients who were being treated by psychiatrists, and consequently, Nakai's “critical period” has been endorsed by many psychiatrists.^36^

Nakai explained how the process progresses sequentially from the “critical period” to the “early remission period” and the “late remission period.” He identified many specific indicators and discovered that there is a great significance in innovative therapeutic engagement in each of these periods. Nakai's findings are supported by meticulous clinical observations and are free from simplifications that stem from simple theorization.

More importantly, close observation of the remission process was conducted with full engagement and concern of the therapist. Without this, the remission process itself would not have been visible, nor would there have been various considerations regarding how to prevent the exacerbation or chronic development of the pathological condition by safely passing through the remission process. Focusing on the remission process in schizophrenia is itself an act of sound optimism. In an era where schizophrenia faced strong pessimistic prejudice as an incurable disease, Nakai's theory of the remission process holds great significance. The meticulous description of remission itself is based on therapeutic (strategic) optimism that seeks to head directly toward potential therapeutics.

## SIGNIFICANCE OF METHODOLOGY

Nakai's theory of the remission process uses a unique approach. This involves the utilization of drawings and progress charts. Pathological phenomena can be determined only by observing the patient's verbal expressions and behavioural characteristics; however, the remission process cannot be determined without increasing the target areas. Nakai refrained from verbally interpreting drawings as in the psychological tests. Rather, he perceived the entire drawing as a series in transition and treated the patient's unspoken inner thoughts as a representation of the drawing itself and succeeded in visually showing changes in the patient's state of mind during the remission process. Nakai is also the person who developed his own drawing therapy technique, the land montage technique (LMT).

Furthermore, the progress chart captures the entire remission process in a single view. Events that fluctuate at various times in many dimensions are also summarized and interpreted more easily. When therapists try to create such a progress chart, they often find that events that they thought were completely unrelated had unexpected relationships. This chart shows the complexities of the various phenomena. Simultaneously, the chart can be used to obtain hints for therapy.

Nakai's method is a naturalistic approach.^36^ According to Nakai,^37^ the Kraepelin‐style concept of the natural course of schizophrenia is a model in which one force moves in one direction, whereas Nakai's model is one in which the two forces of disease onset and natural resilience in two directions. Nakai referred to the work of meteorology in the 1960s, which he applied to unravel the complexity of the chronic disease. If we determine even one aspect of how a certain phenomenon occurs only after a certain phenomenon, then that description is a natural history and a law can be formulated based on that. Therefore, there is a science for unique objects like Mars or Mount Everest. Similarly, the Himalayan peaks are the result of the interaction of meteorological and geological factors, the interaction with the surrounding people is important to the patient's process in schizophrenia. For Nakai, each patient had a Himalayan peak. The reason for paying attention to the natural course is to facilitate the best course of treatment, including the “most humane and gentlest treatment,” a natural course with a “good aftermath,” or simply, the “best course.”

## CONCLUSION

Simply abstracting and understanding Nakai's theory makes it difficult to apply it clinically. Nakai's paper contains many observations and discoveries that support the theory, therefore clinicians who want to learn Nakai's theory should first read his paper repeatedly to relive his observations and discoveries. It is important to cultivate one's own clinical sense and only then will it be possible to apply it to clinical situations.

I began my training as a psychiatrist under Professor Nakai in 1988 and received substantial guidance from him. I also attended his outpatient clinic for approximately 3 years. Professor Nakai's influence on me has been immeasurable. Unfortunately, I received news of his death while I was preparing this article. I would like to thank Professor Nakai for his kindness. May his soul rest in peace.

## AUTHOR CONTRIBUTIONS

Minoru Sugibayashi conceived and prepared this paper alone, but Mayu Shimotamari supported the creation of Figures 1, 2, 3.

## CONFLICT OF INTEREST STATEMENT

The author declares no conflict of interest.

## ETHICS APPROVAL STATEMENT

The ethics approval statement is not applicable.

## PATIENT CONSENT STATEMENT

The patient consent statement is not applicable.

## CLINICAL TRIAL REGISTRATION

The clinical trial registration is not applicable.

## Data Availability

The data that support the findings of this study are openly available at https://doi.org/10.1002/pcn5.96.
